# The use of zebrafish (*Danio rerio*) as biomedical models

**DOI:** 10.1093/af/vfz020

**Published:** 2019-06-25

**Authors:** Tsegay Teame, Zhen Zhang, Chao Ran, Hongling Zhang, Yalin Yang, Qianwen Ding, Minxu Xie, Chenchen Gao, Yongan Ye, Ming Duan, Zhigang Zhou

**Affiliations:** 1 China-Norway Joint Lab on Fish Gut Microbiota, Feed Research Institute, Chinese Academy of Agricultural Sciences, Beijing, China; 2 Key Laboratory for Feed Biotechnology of the Ministry of Agriculture, Feed Research Institute, Chinese Academy of Agricultural Sciences, Beijing, China; 3 Dongzhimen Hospital, affiliated to Beijing university of Chinese Medicine (BUCM), Beijing, China; 4 State Key Laboratory of Freshwater Ecology and Biotechnology, Institute of Hydrobiology, Chinese Academy of Sciences, Wuhan, Hubei, China

**Keywords:** biomedical model, metabolic disorders, zebrafish

**Implications**

- Because of its fully sequenced genome, easy genetic manipulation, high fecundity, external fertilization and rapid development, and nearly transparent embryo, zebrafish are a unique model animal for biomedical research, including studies of biological processes and human diseases.
- Zebrafish have all the main organs involved in the process of metabolism and can be used to study several human metabolic disorders such as nonalcoholic fatty liver disease, type 2 diabetes mellitus, dyslipidemia, and other hepatic diseases.
- With innovation and improvement of molecular techniques, zebrafish will continue to be an important biomedical model in the future.

## Introduction

**Figure 1.**
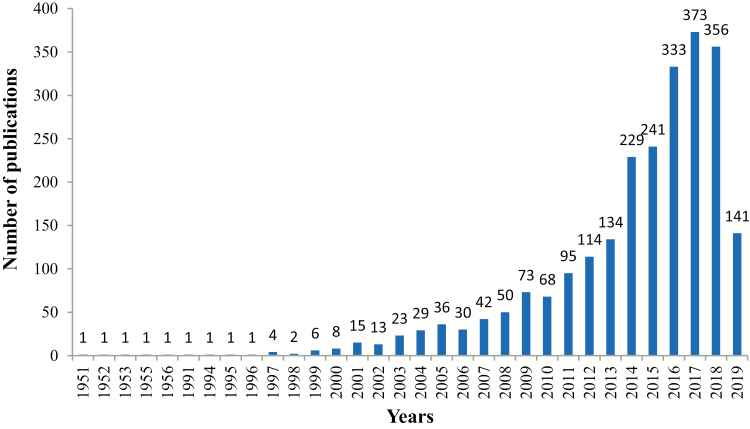
The number of publications in PubMed per year when searching with the keywords “zebrafish” and “Biomedical.”

Various animal species have important roles as experimental models to advance biomedical research. Animal models provide consistency and validity of research results from in vitro studies or studies with rodents. Zebrafish has become a popular animal model for biomedical research. As shown in Figure 1, the number of publications per year on zebrafish as a model for biomedical research has been significantly increasing in recent years. One reason that zebrafish are an important biomedical model is because zebrafish embryos are transparent and they develop outside of the uterus. This unique developmental process allows scientists to study the details of development starting from fertilization and continuing throughout development. Innovation and development of molecular techniques in the later 20th century allowed zebrafish to be used as a model organism in almost all aspects of biology throughout the world. This review focuses on the use of zebrafish as a biomedical model in areas mainly related to diet-induced diseases, metabolic disorders, liver diseases, and intestinal diseases in humans.

## Common Fish Species Used as Model Species

For more than 200 years, scientists used fish as model species with goldfish (*Carassius auratus*) the oldest model species. Goldfish were primarily used for applied studies of aquatic toxicology. Additional fish species have also been used, including zebrafish (*Danio rerio*), goldfish *(Carassius auratus)*, medaka (*Oryzias latipes*), roach (*Rutilus rutilus*), three-spined stickleback (*Gasterosteus aculeatus*), pufferfish (*Takifugu rubripes*), and the swordtail (*Xiphophorus hellerii*) (Ribas and Piferrer, 2014). Every fish species has its unique advantages and disadvantages. For instance, goldfish have been used to study growth, stress, immunology, and reproduction. Medaka fish were the most popular species of fish used to study genetics, reproduction, and development. In recent years, the popularity of zebrafish as a model has increased due to its suitable features for many research areas.

## General Features of Zebrafish

**Figure 2.**
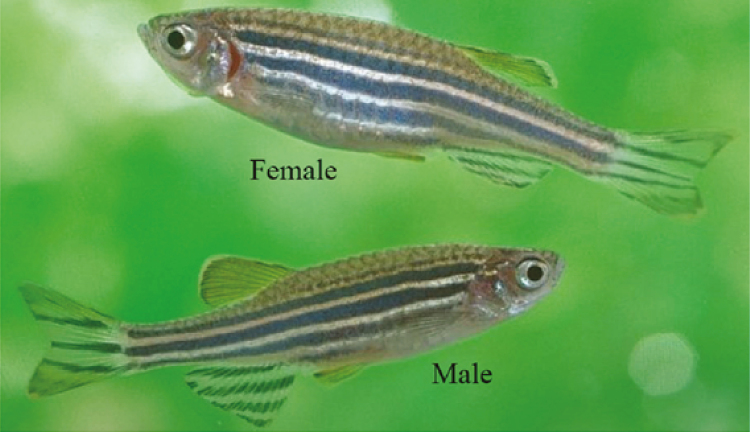
Adult male and female AB strain of zebrafish, adapted from https://www.asianscientist.com/2014/12/in-the-lab/zebrafish-switch-sex/ with minor modification.

*Danio rerio* the Latin name for zebrafish formerly called *Brachydanio rerio* is a small tropical freshwater fish originating in the Ganges River and its tributaries in northern India (Tavares and Santos Lopes, 2013). In the natural habitat, zebrafish are usually found near the bottom of the water to minimize attack by predators. The morphology of male and female zebrafish is shown in Figure 2.

Currently, zebrafish are considered as a suitable model to investigate development, genetics, immunity, behavior, physiology, and nutrition. According to its feeding habits, zebrafish are classified as omnivores and they eat a variety of foods (euryphagous). During experimental trials, scientists use different types and levels of dietary feeds. The same amounts of ingredients are used for adult and larvae zebrafish. Moreover, the feeds and feeding regimes implemented by some laboratories for rearing zebrafish are varied and, in some cases, are implemented without formal evaluation (Castranova et al., 2011; Gonzales and Law, 2013).

In the laboratory, to get reasonable research results, zebrafish should receive the appropriate type and level of dietary nutrients. Most of the time researchers use different commercial diets for zebrafish, but several commercial diets have undefined nutritional composition and may have an effect on experimental results (Gonzales and Law, 2013). In addition, the dietary requirement for larvae and adults are different in the amount and composition of ingredients. In research studies, it is important to use a standard diet with adequate nutritional composition and known ingredients, which promote optimum growth and physiological status of the fish and to minimize the contribution of unintended nutritional effects on experimental results. The following diet formulas (Tables 1 and 2) were developed in our laboratory and give consistent experimental results with zebrafish. We recommend that researchers use these dietary formulas in their studies with zebrafish.

**Table 1.** Dietary formula for zebrafish larvae (5 to 29 d post fertilization)

	Basic feed	High sugar	High fat	Low nitrogen
Raw material (g/100 g diet)				
Casein	46.00	46.00	46.00	32.00
Gelatin	11.00	11.00	11.00	8.00
Dextrin	22.00	31.00	10.00	32.00
Lard oil	–	–	8.00	–
Soybean oil	3.50		8.00	6.00
Cod liver oil	3.50	2.00	4.00	4.00
Soy lecithin	2.00	2.00	2.00	2.00
Lysine	0.37	0.37	0.37	–
VC phosphate	0.10	0.10	0.10	0.10
Vitamin premix^1^	0.20	0.20	0.20	0.20
Mineral premix^2^	0.20	0.20	0.20	0.20
Calcium dihydrogen phosphate	2.00	2.00	2.00	2.00
Choline chloride	0.20	0.20	0.20	0.20
Sodium alginate	4.00	4.00	4.00	4.00
Zeolite powder	4.93	0.93	3.93	9.30
Total	100.00	100.00	100.00	100.00
Proximate composition analysis				
Crude protein (estimated)	48.09	48.09	48.09	33.75
Crude fat (estimated)	9.01	4.01	22.01	12.01
Nitrogen-free extract (estimated)	22.00	31.00	10.00	32.00
Total energy (KJ/g)	15.13	14.75	18.02	15.53

^1^Vitamin premix (g/kg): thiamine, 0.438; riboflavin, 0.632; pyridoxine·HCl, 0.908; *d*-pantothenic acid, 1.724; nicotinic acid, 4.583; biotin, 0.211; folic acid, 0.549; vitamin B_12_, 0.001; inositol, 21.053; menadione sodium bisulfite, 0.889; retinyl acetate, 0.677; cholecalciferol, 0.116; *dl*-α-tocopherol-acetate, 12.632.

^2^Mineral premix (g/kg): CoCl_2_·6H_2_O, 0.074; CuSO_4_·5H_2_O, 2.5; FeSO_4_·7H_2_O, 73.2; NaCl, 40.0; MgSO_4_·7H_2_O, 284.0; MnSO_4_·H_2_O, 6.50; KI, 0.68; Na_2_SeO_3_, 0.10; ZnSO_4_·7H_2_O, 131.93; cellulose, 501.09. (Unpublished data; formulated in our zebrafish laboratory.)

**Table 2.** Dietary formula for zebrafish (1 to 3 mo of age)

	Basic feed	High Sugar	High fat	Low nitrogen
Raw material (g/100g diet)				
Casein	40.00	40.00	40.00	28.00
Gelatin	10.00	10.00	10.00	7.00
Dextrin	28.00	38.00	16.00	38.50
Lard oil	–	–	8.00	–
Soybean oil	6.00	2.00	8.00	6.00
Lysine	0.33	0.33	0.33	–
VC phosphate	0.10	0.10	0.10	0.10
Vitamin premix^1^	0.20	0.20	0.20	0.20
Mineral premix^2^	0.20	0.20	0.20	0.20
Calcium dihydrogen phosphate	2.00	2.00	2.00	2.00
Choline chloride	0.20	0.20	0.20	0.20
Sodium alginate	2.00	2.00	2.00	2.00
Microcrystalline cellulose	4.00	4.00	4.00	4.00
Zeolite powder	6.97	0.97	8.97	11.80
Total	100.00	100.00	100.00	100.00
Proximate composition analysis				
Crude protein (estimated)	42.19	42.19	42.19	29.53
Crude fat (estimated)	6.01	2.01	16.01	6.01
Nitrogen-free extract (estimated)	28.00	38.00	16.00	38.50
Total energy (KJ/g)	14.02	14.18	15.77	13.65

^1^Vitamin premix (g/kg): thiamine, 0.438; riboflavin, 0.632; pyridoxine·HCl, 0.908; *d*-pantothenic acid, 1.724; nicotinic acid, 4.583; biotin, 0.211; folic acid, 0.549; vitamin B_12_, 0.001; inositol, 21.053; menadione sodium bisulfite, 0.889; retinyl acetate, 0.677; cholecalciferol, 0.116; *dl*-α-tocopherol-acetate, 12.632.

^2^Mineral premix (g/kg): CoCl_2_·6H_2_O, 0.074; CuSO_4_·5H_2_O, 2.5; FeSO_4_·7H_2_O, 73.2; NaCl, 40.0; MgSO_4_·7H_2_O, 284.0; MnSO_4_·H_2_O, 6.50; KI, 0.68; Na_2_SeO_3_, 0.10; ZnSO_4_·7H_2_O, 131.93; cellulose, 501.09. (Unpublished data; formulated in our zebrafish laboratory.)

The amount of feed varies across the different growth stages of the fish and is dependent on the stage of growth. From 5 d post fertilization, zebrafish larvae are mostly fed zooplanktons such as paramecium and rotifers and young larvae can be fed with artificial food up to 100 μm in size or live feed. For adult fish, the size of the dry food can range from 300 to 400 μm (Avdesh et al., 2012). The size of the dry food can increase with increasing size of the fish. The commonly practiced feeding ratio of zebrafish is about 4% of its bodyweight. Overfeeding may increase the concentration of nitrate in the water and affect the physiology of the fish. In addition, overeating may cause death of the fish.

## Why Do Zebrafish Make Such Good Animal Models?

The criteria to select animal models for biomedical research are directly related to the final goal of the research. The use of zebrafish as a biomedical model was suggested by George Streisinger and colleagues at the University of Oregon, who launched the modern era for zebrafish in the field of biomedical research (Clark and Ekker, 2015). Zebrafish are popular animal models because they have numerous advantages over other species. The most advantageous features of zebrafish are a fully sequenced genome, easy manipulation of its genome, high fecundity, short generation time (about 3 mo), rapid embryonic development (24 hr), and external fertilization. The translucent zebrafish embryo allows study of the different developmental stages starting from the early stage of embryogenesis. In addition, zebrafish embryos form complete organ systems, including heart, intestine and blood vessels within 48 hr after fertilization. More than 10,000 mutants in protein-coding genes have been generated (Howe et al., 2013) and several transgenic lines of zebrafish have been made to study human diseases. The availability of multiple strains of zebrafish is another important advantage of this species. In addition, it is also very affordable to maintain a large number of zebrafish in a relatively small amount of laboratory space. Although zebrafish require relatively easy management, special attention must be paid to ensuring a healthy diet and adequate water quality to optimize fish health and growth. While there are several strains of zebrafish in the world, the most widely used strains in biomedical research are AB, Casper, Ekkwill, Nadia, Wild Indian Karyotype, wild-caught, and Tubingen. According to the ZFIN website, more than 800 biological laboratories around the world conduct basic and applied research with zebrafish (https://zfin.org/search?q=Zebrafish+laboratories&category). Many of these laboratories use zebrafish to study human diseases, including neural disorders, cancer, infectious diseases, cardiovascular diseases, kidney diseases, diabetes, blindness, deafness, digestive diseases, hematopoiesis, and muscle disorders.

Mutant zebrafish have been established by knocking out or knocking in specific genes. These alterations create novel biomedical models. For example, if the patient has a disease related to metabolism, different mutations in zebrafish genes related to metabolism can be made and then changes in gene expression can be monitored using different molecular techniques. The short generation time of zebrafish makes it difficult to produce stable transgenic adults or homozygous mutant embryos, which usually requires about 4 months. Recently, scientists have developed many technologies to expedite the transgenic process (Burger et al., 2016). The presence or absence of genomic duplication events in zebrafish makes it complicated to study some human diseases such as diabetes mellitus. Zebrafish are also important for developing new therapies or screening novel drugs to treat or prevent human diseases.

Even though zebrafish are an important biomedical model, they have some limitations, including the dissimilarity of some organs like the respiratory system and the reproductive system. Thus, it is difficult to use zebrafish as a model for respiration or reproduction in humans. In addition, because zebrafish live in an aquatic habitat, screening of some water soluble drugs in zebrafish is another limitation.

## Zebrafish as a Model for Metabolic Diseases

**Figure 3.**
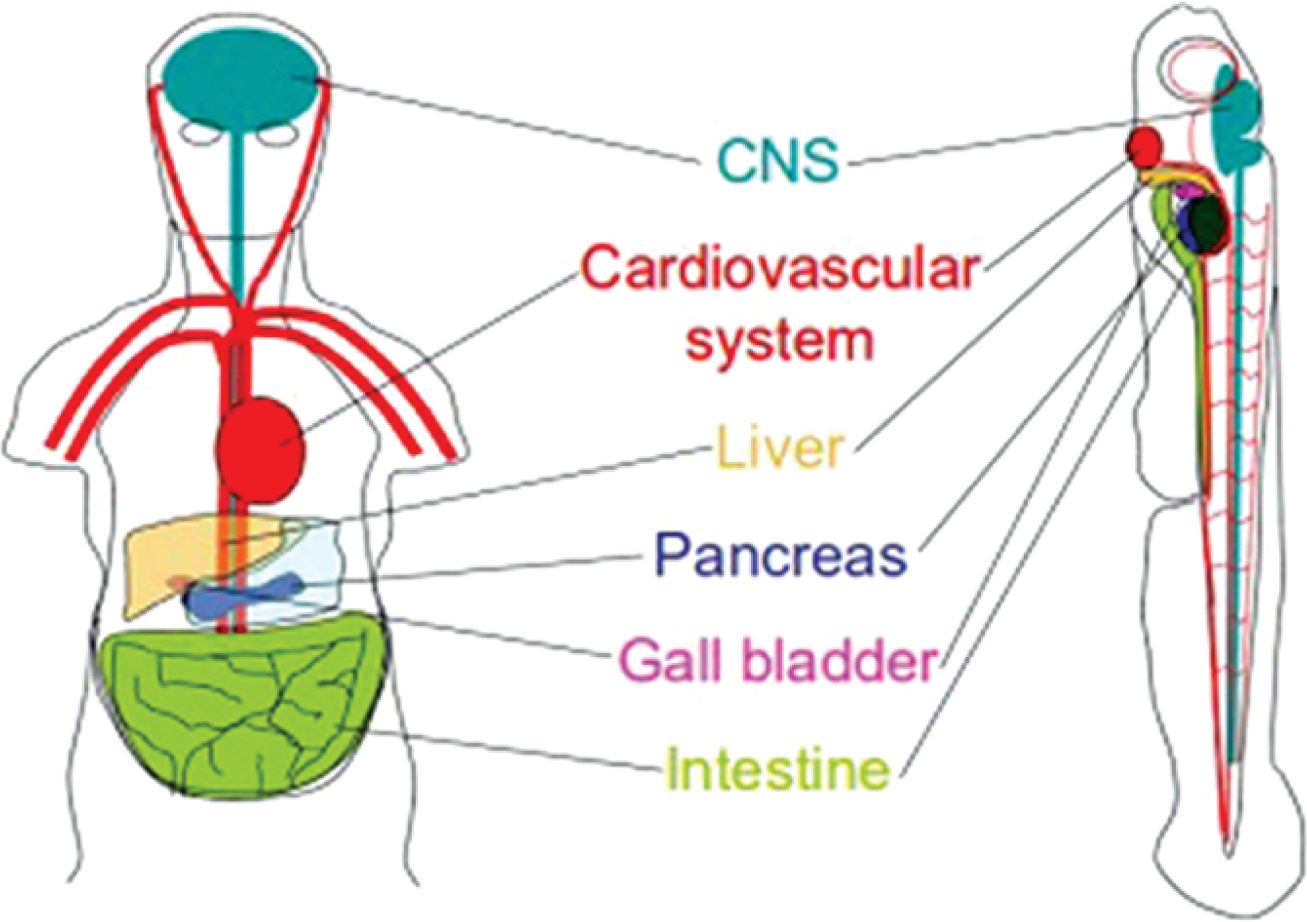
Some of the conserved organ systems between zebrafish and humans (adapted from http://www.intl.upm.edu.my/article/zebrafish_replace_lab_rat-30977 with minor modification).

There are several examples of human diseases that have been successfully modeled in zebrafish such as Duchenne muscular dystrophy, human melanoma, acute lymphoblastic leukemia, polycystic kidney disease, nephronophthisis, acute kidney injury, Parkinson’s disease, Huntington’s disease, Alzheimer disease, myocardial infarction, and some metabolic diseases. As shown in Figure 3, in addition to genomic similarity, the presence of conserved organs and organ systems between human and zebrafish contributes to development of a number of successful models of human diseases.

We will focus on the common human metabolic diseases successfully modeled in zebrafish, including obesity, type 2 diabetes mellitus, nonalcoholic steatohepatitis, and atherosclerosis. Disturbance of the normal process of converting food to energy in the cell results in different metabolic disorders. Even though zebrafish and humans have differences in basic nutrient requirements, different metabolic mechanisms may not be needed. To keep the balance between the production and utilization of energy several organs are involved, including the brain, intestines, liver, skeletal muscle, and adipose tissue. Whole animal models are needed to study the entire process of metabolism. Zebrafish are an appropriate model to study metabolic dysfunction because they have all the organs involved in energy homeostasis and metabolism including appetite and insulin regulation and a lipid storage system which is conserved with that found in humans (Nishio et al., 2012).

A report from World Health Organization indicated that, of the metabolism-related human diseases, cardiovascular disease is currently the most predominant fatal disease (Lozano et al., 2012). Obesity (Ng et al., 2014), type 2 diabetes mellitus, and nonalcoholic fatty liver disease (LaBrecque et al., 2014) increase the risk of cardiovascular disease. Because zebrafish and humans have similar metabolic organs (including the digestive organs, adipose tissue, and muscle), zebrafish are a popular model to study metabolic disorders. In addition, the availability of several new tools and approaches such as talens, CRISPR/Cas9 (Wu et al., 2018), compound treatment (Poureetezadi et al., 2016), mass spectrometry-based polar metabolomics and lipidomics (Zhang et al., 2018), and in vivo imaging of fluorescent dyes (Minchin et al., 2018) make it possible to investigate the molecular mechanisms of metabolic processes in zebrafish.

Researchers have also used zebrafish as a model organism to study different types of metabolic diseases such as congenital errors of metabolism, hyper- and hypothyroidism, disorders of the hypothalamus–pituitary–adrenal axis, dysregulation of the circadian clock, and cancer metabolism (Gut et al., 2017). In this review, our emphasis will be on diet-induced metabolic disorders.

## Zebrafish as a Model Animal for Diet-induced Obesity

**Figure 4.**
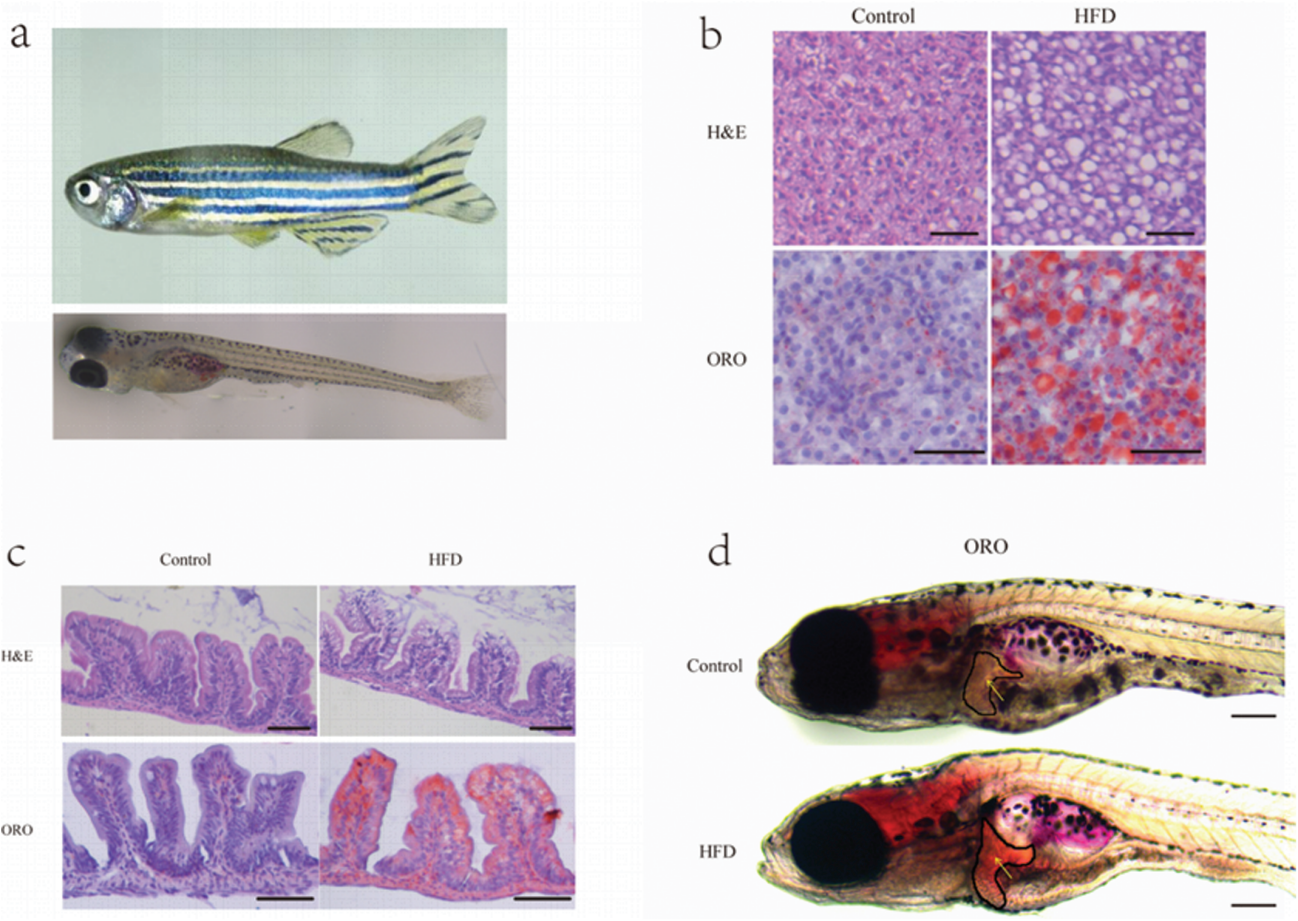
High-fat diets (HFD) induced hepatic steatosis in adult and larval zebrafish. (a) Adult zebrafish (1 mo old) and larval zebrafish (5 d post fertilization). (b) Representative liver histology image by Haemotoxylin and Eosin (H&E) staining and oil red O (ORO) staining of adult zebrafish fed with a control diet or HFD for 4 wk. The scale bar is 50 μm. (c) Representative intestinal histology image by H&E staining and ORO staining of adult zebrafish fed with a control diet or HFD for 4 wk. The scale bar is 100 μm. (d) Representative image of whole-mount ORO staining in zebrafish larvae fed control diet and HFD for 7 d. The scale bar is 200 μm. (Unpublished data from our zebrafish laboratory.)

Utilization of zebrafish in diet-induced obesity studies was first developed by Oka et al. (2010) by feeding adult zebrafish *Artemia nauplii*. In these studies, the fish showed increased body mass index, developed hepatic steatosis, hypertriglyceridemia, and dysregulation of some lipid metabolism genes. Chen et al. (2018) fed zebrafish a diet of high cholesterol, which resulted in increased body weight, increased triglyceride levels, and lipid deposition in the liver. Over nutrition of zebrafish with high fat from different sources or cholesterol also lead to hyperglycemia and ectopic lipid accumulation, increased body weight, increased adipose tissue, cardiovascular overload, and steatosis (Forn-Cuní et al., 2015). Landgraf et al. (2017) used zebrafish to compare the result of overfeeding with normal and high-fat diets on obesity development. They concluded that both diets showed an increase in adipose tissue and the fish fed the normal fat diet developed obesity, but these fish were metabolically healthy. The other fish fed a high-fat diet were unhealthy. Similar with the above findings, in our laboratory, we also found that larvae and adult zebrafish fed a high-fat diet developed hepatic steatosis as shown in Figure 4. In zebrafish, diet-induced obesity is also used to estimate the type of food and effect of nutrient compounds on development, testing, and discovering different drugs to prevent or treat obesity and by altering fat metabolism. The diet-induced obesity zebrafish model overfed with *Artemia* shares common pathophysiological pathways with mammalian obesity and can be used to identify putative pharmacological targets of human obesity (Oka et al., 2010). Therefore, the diet-induced obesity approach allows us to understand the disease in the context of systematic obesity, hence mimicking the most common process occurring in humans affected by this condition.

## Zebrafish as Model for Glucose Metabolism and Type 2 Diabetes Mellitus

The main cause for development of diabetes mellitus is the failure of pancreatic β-cells to produce insulin, which leads to insulin deficiency. These functions and processes are conserved between zebrafish and humans. Zebrafish exposure to hypercaloric and high-fat diets quickly induces obesity and obesity‐related disease, and activates metabolic pathways very similar to their human counterparts. If glucose is available in the diet, insulin is produced by the pancreas, and gluconeogenesis is inhibited through the down-regulation of genes involved in the pathway. In the absence of glucose in the bloodstream, gluconeogenesis is induced by the action of glucagon. Capiotti et al. (2014) revealed that zebrafish immersed in a high-glucose solution (111 mM) for 14 d were able to increase by 41% froctosamine (glycated protein) levels from the eyes, decreased amounts of mRNA for insulin receptors in muscle, and developed hyperglycemia. Zang et al. (2017) developed a zebrafish model of type 2 diabetes mellitus by overfeeding a high-calorie diet (408 calories per fish per day). Using gene expression profiling in the liver and pancreas, a common pathway for development of type 2 diabetes mellitus was seen between zebrafish and humans. The relationship between age and type 2 diabetes mellitus was developed by Connaughton et al. (2016) and revealed that young zebrafish (4 to 11 mo olds) developed hyperglycemia slower than old zebrafish with increasing concentrations of glucose. The glucose concentration of homeostasis organs can be increased by immersing zebrafish embryos in a glucose solution. Gleeson et al. (2007) showed that immersion of adult zebrafish in a 1% glucose solution for 24 hr increase blood glucose up to 400 mg/dL. The two transgenic models of insulin resistance established by Zang et al. (2017) were skeletal muscle insulin resistance achieved by transgenic expression of a dominant-negative IGF-I receptor in skeletal muscle. In the second model, insulin resistance was attained via liver--specific knockdown of the insulin receptor gene using CRISPR/Cas9 (Yin et al., 2015). These results revealed that zebrafish are a suitable model to study glucose-induced human disease. Marín-Juez et al. (2014) also developed a zebrafish model for hyperinsulinemia by injecting human recombinant insulin in zebrafish larvae. These studies demonstrated upregulation of the negative immune modulator protein tyrosine phosphatase non receptor type 6 in insulin-resistant larvae. Recent research results of Yang et al. (2018) showed that mutant zebrafish with a knockout in insulin receptor a and b genes when fed a high-carbohydrate (41%) diet showed hyperglycemia, reduced growth hormone signaling, increased visceral adiposity, and fatty liver development, which are similar signs to the human lipodystrophy disease. The glucose level in zebrafish can be measured using two hand-held glucose meters designed for use in humans with diabetics (Eames et al., 2010). Additionally, fasting for performing postprandial glucose and intraperitoneal glucose tolerance tests can be used. There are several methods of measuring insulin levels in zebrafish, including measuring the insulin mRNA expression level by q-PCR (Michel et al., 2016), insulin antibody for immunostaining (Kimmel et al., 2015), or semi-quantitative dot-blot (Olsen et al., 2012). Insulin sensitivity can also be assessed by intraperitoneal injection of insulin in hyperglycemic zebrafish (Capiotti et al., 2014).

## Zebrafish as Model for Dyslipidemia and Atherosclerosis Diseases

Increasing the level of cholesterol, triglycerides, or high-density lipoprotein cholesterol resulted in dyslipidemia, and in turn, led to development of atherosclerosis. Since the nutritional requirements of zebrafish are known, several researchers established different models by changing the standard diet (such as feeding zebrafish a high-fat diet to develop obesity, hyperglycemia, and dyslipidemia) to induce metabolic stress on the fish. The histopathological changes showed by zebrafish fed a high level of cholesterol are very similar with the symptoms shown in human atherosclerosis (Fang and Miller, 2012). Formulation of a high-cholesterol diet is also important for the study of dyslipidemia (Oka et al., 2010). Miyares et al. (2014) described lipid and lipoprotein metabolism using the zebrafish embryo yolk metabolism stages and concluded that incorporation of exogenous fatty acids into the circulatory system was dependent on lipoprotein production in the system.

## Zebrafish as a Model for Nonalcoholic Fatty Liver Disease and Other Liver Disorders

Nonalcoholic fatty liver disease is not related to overconsumption of alcohol. It is the accumulation of excess fat in the liver, and this can lead to steatosis, steatohepatitis, fibrosis, corrihosis, and hepatocellular carcinoma. This disease can develop and be associated with insulin resistance, high-fat diets, drug-induced liver injuries, and metabolic syndromes. Several research results show that zebrafish also develop hepatic steatosis when exposed to hepatotoxic chemicals, fasting and excessive dietary fat, cholesterol, or carbohydrate. These mechanisms are similar in zebrafish and humans. Interestingly, publication of the first paper on zebrafish development (Roosen, 1937) investigated the effect of different toxins, alcohol, and different levels of carbohydrate or fat diets on zebrafish embryos, larvae, and adult developmental stages. The application of toxins to the fish tank is a simple technique and this technique makes zebrafish a popular model to study chemical screening mechanisms.

The zebrafish liver resembles the human liver in cellular structure, function, and genetics. This observation led investigators to use zebrafish to study the detailed embryological and genetics associated with development of the human liver, as well as liver disorders and potential therapies for liver diseases. Development of liver tumors in zebrafish using carcinogenic substances and comparison with gene expression in tumors of human livers first pointed to the importance of zebrafish as an appropriate biomedical model. Tonin et al. (2018) showed that zebrafish immersed in 6% fructose lead to the formation of hepatic steatosis in a manner similar to the symptoms shown in humans fed a high-carbohydrate diet. Using a differential feeding strategy, Yang et al. (2019) showed that over feeding resulted in development of fatty liver and hastened the carcinogenic process. In addition, the hormone leptin, which is responsible for obesity, was unregulated in the oncogenic and overfed zebrafish. They also found that, by downregulating leptin signaling, it is possible to reduce the muscle wasting phenotype. Development of a mutated gene *foie gras* in zebrafish initiated scientists to study development of hepatic steatosis and the associated molecular mechanisms. In addition, development of *gonzo* mutant zebrafish showed that development of alcohol-induced hepatic steatosis was mediated by sterol response element binding protein transcription factors (Passeri et al., 2009). Shimada et al. (2015) applied transcriptomic and proteomic methods using a model of diet-induced obesity in the liver of zebrafish to isolate genes responsible for the formation of hepatic steatosis. In these studies, fatty acid binding protein 3 and transcription factors (E2F) were upregulated in hepatic steatosis zebrafish. Howarth et al. (2013) developed two models using zebrafish to investigate either tunicamycin- or ethanol-provoked steatosis which leads to liver failure. They prevented ethanol-induced steatosis by blocking activation of sterol response element binding proteins using mutant zebrafish. In these studies, even without lipid accumulation, hepatocyte dysfunction occurred. Recent research from Imran et al. (2018) using zebrafish larvae to test the involvement of membrane remodeling in hepatotoxicity showed that co-exposure of obese zebrafish larvae to benzo[a]pyrene and ethanol induced in vivo hepatotoxicity through membrane remodeling. This result led scientists to develop a therapy for nonalcoholic fatty liver disease and associated risk factors.

## Zebrafish as a Model for the Study of Intestinal Diseases and Host–Microbe Interactions

The intestine of zebrafish is a long tube like structure, which has been divided into the intestine bulb, mid-intestine, and posterior intestine that folds twice in the abdominal cavity. The absorptive enterocytes, goblet cells, and endocrine cells are the three cell types that have differentiated from the intestine epithelium. Since innovation of forward genetic screening techniques, many scientists have used zebrafish as a model to study the physiology, function, and diseases of the human intestine. The entire intestinal track opens at 6 d post fertilization and at this time larvae start to feed on small aquatic animals (Brugman, 2016). At this stage of development, the intestine of the fish is easily visible and its morphology can be observed with a microscope. Because of its transparent body, many researchers have developed a zebrafish model of intestinal inflammation. Ji et al. (2018) developed a zebrafish model to evaluate how bioactive compounds are taken up by the intestine. They concluded that bioactive compounds are able to cross the intestinal mucosal barriers and pass through the lamina propria to reach the muscle. Arias-Jayo et al. (2018) showed that zebrafish fed a high-fat diet of 10% (w/w) cocoa butter added to the normal diet resulted in intestinal inflammation via activation of NF-κβ. The intestinal barrier was also damaged and there was an increase in mucin production by goblet cells. Oehlers et al. (2011a) developed a model with zebrafish embryos infected with salmonella, and showed that depletion of the bacterial detector proteins NOD1 and NOD2 reduced expression of the dual oxidase in the intestinal epithelial. This also weakened the ability of the fish to reduce the intracellular burden of bacteria. Overall, this finding was a good model for Crohn’s disease in humans.

Zebrafish have also been used to study host–microbe interactions in the digestive system. Recent studies of the intestinal microbiome in zebrafish with a mutation in gene *myd88* demonstrated that changes due to the microbiome in the body (especially the intestinal leukocytes) are dependent on the immune adaptor gene *myd88* (Koch et al., 2018). Raising germ-free zebrafish to investigate the effect of microbiota on the innate immune system has also been studied (Kostic et al., 2013) and the contribution of gut microbes on fatty acid absorption was studied by Semova et al. (2012). In these studies with zebrafish, the fish with microbes in their gut had increased fatty acid absorption, higher accumulation of fats in the liver and the body when compared with germ-free zebrafish. The gut microbiota of human and zebrafish are different. Valenzuela et al. (2018) showed that germ-free zebrafish larvae can be colonized by human gut microorganisms, such as *Clostridioides difficile* and *Bacillus*. This result opened an interesting area to study interactions between these microorganisms and the host. The role of the gut microbiota on host biology is similar between zebrafish and mammals and, in both species, intestinal microbiota participate in the education of the immune system, maturation of the gut, and promotion of nutrient metabolism in the host (Bates et al., 2007).Therefore, zebrafish are an important model to further explore intestinal diseases and related aspects of gut biology.

## Conclusion

Zebrafish are an important biomedical model in every aspect of biology. Zebrafish have several suitable features for developmental, physiological, and genetic studies including external fertilization and the transparent nature of embryo. The large degree of functional conservation of morphology, genetics, and physiology between zebrafish and humans makes zebrafish an attractive model for several human disorders and development of potential therapies for humans. Advancement of nanotechnologies and molecular techniques also contributes to the use of zebrafish to study different diseases in humans. In this review, we emphasized some biomedical areas where zebrafish are a popular model to investigate the mechanisms and processes associated with metabolic diseases, including diet-induced obesity, type 2 diabetes mellitus, dyslipidemia and atherosclerosis, liver-related diseases, and intestinal diseases. Scientists have also used zebrafish to develop new therapies to treat and prevent these important human diseases.

## Acknowledgments

This work was supported by the National Natural Science Foundation of China (31872584, 3180131599, 31702354, 31602169, 31672294, 31572633), the Beijing earmarked fund for Modern Agro-industry Technology Research System (SCGWZJ 20191104-4), and Innovation Capability Support Program of Shaanxi (2018TD-021).

## About the Authors

**Tsegay Teame** is a PhD student in the laboratory of Dr. Zhigang Zhou and the Innovation Team of Aquatic Animal Feed at the Feed Research Institute, Chinese Academy of Agricultural Science, Beijing, China. His research focuses on the effects of high-fat diet on the gut microbiota of zebrafish. He received his M.Sc. in Aquaculture and Fisheries from Ambo University, Ethiopia.

**Zhen Zhang** received his PhD from the Chinese Academy of Agricultural Sciences, Beijing, China. He is currently a postdoctoral trainee in the laboratory of Dr. Zhigang Zhou and the Innovation Team of Aquatic Animal Feed at the Feed Research Institute, Chinese Academy of Agricultural Sciences, Beijing, China.

**Chao Ran** received his PhD from Auburn University, Alabama, USA. He is currently associate professor in the Feed Research Institute, Chinese Academy of Agricultural Sciences, Beijing, China.

**Yalin Yang** received her PhD from the Chinese Academy of Agricultural Sciences, Beijing, China and is currently deputy researcher of Biochemistry and Molecular Biology in the Innovation Team of Aquatic Animal Feed, at the Feed Research Institute, Chinese Academy of Agricultural Sciences, Beijing, China.

**Qianwen Ding** received her MSc from the Chinese Academy of Agricultural Sciences, Beijing, China. She is currently a research assistant in the Key Laboratory for Feed Biotechnology of the Ministry of Agriculture at the Feed Research Institute, Chinese Academy of Agricultural Sciences, Beijing, China.

**Mingxu Xie** received her MSc from Dalian Ocean University, Dalian, China. She is currently a research assistant in the Key Laboratory for Feed Biotechnology of the Ministry of Agriculture at the Feed Research Institute, Chinese Academy of Agricultural Sciences, Beijing, China.

**Hongling Zhang** received her PhD from the Chinese Academy of Agricultural Sciences, Beijing, China. She is currently a postdoctoral trainee in the laboratory of Dr. Zhigang Zhou, China-Norway Joint Lab on Fish Gastrointestinal Microbiota, Feed Research Institute at the Chinese Academy of Agricultural Sciences, Beijing, China.

**Chenchen Gao** received her BSc from the Yunnan Normal University, Yunnan, China. She is currently a research assistant in the Key Laboratory for Feed Biotechnology of the Ministry of Agriculture, Feed Research Institute, Chinese Academy of Agricultural Sciences, Beijing, China.

**Ye Yong’an** is currently the chairman of the Committee of Hepatology of the World Federation of Chinese Medicine Societies. As one of the most prominent hepatologists in the field of viral hepatitis B in China, he has been dedicated to the treatment of chronic liver diseases with integrative approaches of traditional Chinese herbal medicines plus modern therapies for decades. Funded by the National Health Commission of the P.R. China, he has presided over five large-scaled clinical studies nationwide, from which he had established a series of innovative therapeutic protocols for chronic hepatitis B and made a significant contribution to the control of viral hepatitis B in P.R. China. Moreover, he is also well known in treating refractory diseases such as liver cirrhosis, and gastrointestinal cancers with Chinese herbal medicines.

**Min Duan** received his PhD from the Institute of Hydrobiology, Chinese Academy of Science. He was a postdoctoral fellow in the East China Sea fisheries Research Institute, Chinese Academy of Fishery Science/Shanghai Ocean University. His research interest is in fish ecology. Currently, he is working as an associate researcher at the Institute of Hydrology, Chinese Academy of Science, Hubei, China

**Zhigang Zhou** is the Director of the Aqua Feed Innovation Research Team, Feed Research Institute of Chinese Academy of Agricultural Sciences, Head of the China-Norway Joint Lab on Fish Gut Microbiota, and Associate Chairman of the International Research Consortium on Gastrointestinal Microbiota in Aquatic Animals. His current research interests focus on understanding the interaction between gut microbiota and the piscine host. He is also interested in microbial products to support sustainable aquaculture.
